# Research on the Combination of Firefly Intelligent Algorithm and Asphalt Material Modulus Back Calculation

**DOI:** 10.3390/ma15093361

**Published:** 2022-05-07

**Authors:** Runmin Zhao, Jinzhi Gong, Yangzezhi Zheng, Xiaoming Huang

**Affiliations:** 1 School of Transportation, Southeast University, Nanjing 211189, China; 230228857@seu.edu.cn (R.Z.); gjz273664@126.com (J.G.); zyzz@seu.edu.cn (Y.Z.); 2 Transportation Bureau of Jinhu, Huaian 211600, China; 3 National Demonstration Center for Experimental Education of Road and Traffic Engineering, Southeast University, Nanjing 211189, China

**Keywords:** back calculation of modulus, Falling Weight Deflectometer (FWD), firefly optimization algorithm, RIOHTrack

## Abstract

The modulus of asphalt pavement material is a necessary parameter for the design, strength measuring and stability evaluation of asphalt pavement. To get more precise test data for asphalt pavement material modulus, a new modulus back calculation method is proposed in this article, named as the Firefly Asphalt Back Calculation Method (FABCM). This novel method uses the firefly optimization algorithm, which is a kind of particle swarm intelligence algorithm imitating the information transfer process among fireflies. To demonstrate the reliability and stability of FABCM, and to study the feasibility of multi-parameter modulus back calculation methods, this article used theoretical deflection curves calculated by BISAR3.0 and the actual measurement data of deflection curves and vertical pressures on the subgrade top surfaces on the full-scale test circular track in the Research Institute of Highway, Ministry of Transport (RIOHTrack) to conduct a modulus back calculation. The results show that FABCM only takes 0.5–1 s for each calculation, and the back calculation errors in the verification of FABCM are mostly smaller than 1%, which means that the firefly optimization algorithm was modified effectively in this article. Moreover, this article also indicates some key factors influencing the accuracy of modulus back calculation, and several reasonable suggestions to the application of modulus back calculation.

## 1. Introduction

The modulus of asphalt pavement material is an important parameter in asphalt pavement design, construction and maintenance, and both indoor and outdoor test methods, which can get the precise modulus of both bitumen and asphalt mixtures, are important in asphalt concrete research. For indoor tests, there are tensile creep tests [1], a dynamic mechanical analyzer (DMA) [2] and the M2F trapezoid beam loading facility [3] to determine the dynamic modulus of asphalt mixture, dynamic sheer rheometer (DSR) tests to determine the dynamic shear modulus of bitumen [4,5,6], and a simple performance tester (SPT) [7] to determine the dynamic shear modulus of asphalt mixture, etc. As for the outdoor methods, the back calculation of modulus based on the deflection curve obtained by a Falling Weight Deflectometer (FWD) is also widely used.

In 1968, Scrivner et al. [8] developed the first modulus back calculation method based on FWD deflection curves, and then designed and made the first Nomogram Abac. The modulus back calculation methods in the early stage are mostly based on regression analysis methods. For example, Sun et al. [9] determined the regression equations of subgrade modulus and surface layer modulus with 792 deflection curves of two-layer asphalt pavement structures. Yang et al. [10,11] developed a logarithmic regression equation with 1000 samples, and then conducted modulus back calculation on both the subgrade and surface layer. Although the modulus back calculation methods based on regression equations were developed earliest, it was proved that this kind of method could hardly give satisfying results in practical applications [12].

To pursue the smallest error between the theoretical deflection curve and measured deflection curve, nonlinear programming methods were used on modulus back calculations [13]. Typical modulus back calculation methods, based on nonlinear programming methods, are the “DEF” series (BISDEF and CHEVDEF) of programs by the U.S. Army Corps of Engineers, MODCOMP (Moduli Computation) by Irwin, WESDEF by Water-ways Experiment Station et al. [14]. To guarantee the convergence of nonlinear programming methods, it is usually necessary to give a strict modulus value range, which could restrict the application of these algorithms [15].

With the development of computer and data storage technology, the back calculation methods based on database search methods were also proposed, like the MODULUS developed by Uzah et al. [16,17]. In this kind of method, a direct search and interpolation algorithm are used to determine the modulus combination, which meets the deflection curve accuracy requirements [11]. In the research of Wang et al. [18], big errors appeared when the back calculated modulus combination was not included in the database, which meant the universality of back calculation methods based on the database search method was not satisfactory.

Furthermore, Cheng et al. [19] proved that the modulus back calculation methods based on the artificial neural network (ANN) are more stable and accurate compared with other methods. Due to the strong ability to conduct nonlinear dynamic processing, and the ability to adaptively build mapping relations between the input and output parameters, ANN has a strong fault tolerance for incomplete samples or uncertain samples containing fuzzy and random data, and is also suitable for big data processing [20]. Zaman et al. [21], Huang et al. [22], Wang et al. [23], Yang et al. [24,25,26,27], and Khazanovich et al. [28,29,30] proved that the rebound modulus of asphalt pavement materials could be calculated precisely with ANN. Moreover, another kind of intelligence-based algorithm used in the modulus back calculation is the swarm intelligence algorithm, which consists of population initializations, individual updates and group updates [31]. Fwa et al. [32], Himeno et al. [33], and Zha et al. [34] tried to apply the genetic algorithm in the modulus back calculation, which shows that the back calculation speed is usually slow due to the need for plenty of genetic calculations to obtain stable results; otherwise it is hard to reach a balance between calculation speed and the avoidance of being premature.

There are three factors which affect the accuracy of pavement modulus back calculation: the differences between a theoretical model and the actual pavement structure; the inherent defects of mathematical optimization algorithm; and the errors of input parameters [35,36]. To reduce the inherent defects of mathematical optimization algorithm, and to reduce the influence of single parameter errors on back calculation results, the Rosenbrock search method and the Gaussian perturbation strategy were introduced in this paper to modify the firefly optimization algorithm. Results of back calculation conducted both on theoretical deflection curves and measured deflection curves on RIOHTrack were analyzed by this new firefly asphalt back calculation method to verify whether the new algorithm has better practicality.

## 2. Materials and Methods

### 2.1. Material and Structure Parameters

#### 2.1.1. Materials and Structures Used in the Theoretical Deflection Curve Calculation

To evaluate the influences of load levels and distribution of deflection curve points on back calculation results, 6 typical asphalt pavement structures including 2 flexible pavement structures, 2 semi-rigid pavement structures and 2 composite pavement structures were selected in this study. All the 6 asphalt pavement structures are simplified into the structures of 3 or 4 layers, which could be seen in Table 1:

#### 2.1.2. Materials and Structures Used in the Actual Deflection Curve Measurement

**Figure 1:**
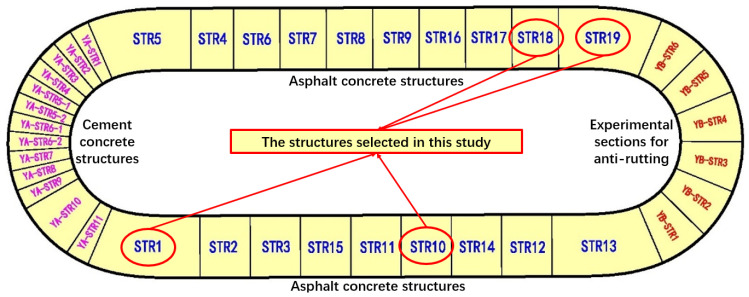
The structures in RIOHTrack.

As the first full-scale pavement testing loop in China, RIOHTrack was built in Tongzhou, Beijing. There are 19 kinds of asphalt pavement structures and 13 kinds of cement concrete pavement structures in RIOHTrack, as is shown in Figure 1:

There are a total of 4 kinds of pavement structures in RIOHTrack, which are namely STR1, STR10, STR18 and STR19, and were selected by investigating the most typical asphalt pavement structures around the world. All 4 structures were selected to conduct modulus back calculation by FABCM. STR1 consists of a thin asphalt concrete surface layer, a cement treated macadam base layer and cement treated soil subbase layer; STR10 consists of an asphalt concrete surface layer, a graded macadam base layer and a cement treated macadam subbase layer; STR18 consists of an asphalt concrete surface layer and a graded macadam base layer; STR19 consists of a thick asphalt concrete surface layer and a thin cement treated macadam base layer. To simplify the structures, the principle of equivalent modulus [37] (as is shown in Equations (1) and (2)) was used before the back calculation procedure:

(1) $$h_{i}^{*}=h_{i1}+h_{i2}$$

(2) $$E_{i}^{*}=\frac{E_{i1}h_{i1}^{3}+E_{i2}h_{i2}^{3}}{{\left(h_{i1}+h_{i2}\right)}^{3}}+\frac{3}{h_{i1}+h_{i2}}{\left(\frac{1}{E_{i1}h_{i1}}+\frac{1}{E_{i2}h_{i2}}\right)}^{-1}\;$$

where $h_{i}^{*}$ refers to the equivalent thickness (mm); $E_{i}^{*}$ refers to the equivalent modulus (MPa).

The simplified structures of STR1, STR10, STR18 and STR19 are shown in Table 2:

### 2.2. The Firefly Asphalt Back Calculation Method (FABCM)

#### 2.2.1. The Modified Firefly Optimization Algorithm

The firefly optimization algorithm is simulated according to the information transfer process among fireflies in nature [38,39,40,41]. The key factor of the firefly optimization algorithm is that fireflies with a low brightness are always attracted by fireflies with a high brightness and update their positions according to the position update equation. Furthermore, the firefly with the highest brightness will update its position immediately. After that, all fireflies will update their brightness and move continually according to the attraction rules.

The three basic hypotheses of the firefly optimization algorithm are as follows:

1. It is assumed that all fireflies are equally attracted to each other, and the less luminous fireflies are attracted to and move towards the brighter ones;
2. The attraction between two fireflies is only related to the distance between them and their luminous intensity. The attraction decreases with the increase in distance. Low intensity means less attraction to other fireflies;
3. The luminous intensity is determined by the objective equation.

The distance $r_{ij}$ between firefly i and firefly j could be determined as:

(3) $$r_{ij}=\|X_{i}-X_{j}\|=\sqrt{\overset{d}{\underset{k=1}{\sum }}{\left(x_{i,k}-x_{j,k}\right)}^{2}}$$

where d refers to the dimension of the decision variable.

The attraction β of firefly could be expressed as follows:

(4) $$\beta =\beta_{0}e^{-\gamma r_{ij}^{2}}$$

where $\beta_{0}\in \left[0,1\right]$, refers to the attraction when $r_{ij}=0$; and γ∈[0,10], refers to the fluorescence absorption coefficient.

The moving and updating of firefly positions could be described as: fireflies are attracted to brighter fireflies and shift their positions:

(5) $$x_{i}^{t+1}=x_{i}^{t}+\beta_{0}\cdot e^{-\gamma \cdot r_{ij}^{2}}\left(x_{j}^{t}-x_{i}^{t}\right)+\alpha \cdot \left(rand-0.5\right)$$

where $x_{i}^{t}$ refers to the position of firefly i at the time t; and α∈[0,1] refers to the random step, rand~U(0,1) is a random number.

As for the brightest firefly, it will fly randomly:

(6) $$x_{best}^{t+1}=x_{best}^{t}+\alpha \cdot \left(rand-0.5\right)$$

where $x_{best}^{t}$ is the optimal individual position in the firefly population of generation t.

Firefly optimization algorithm has plenty of advantages. For instance, due to the individual searching and the sensing ability of each firefly, it usually does not lead to local convergence, and it can be carried out concurrently. However, the traditional firefly optimization algorithm also faces some problems. As a result of the limited search range of each firefly, the firefly optimization algorithm could fall into local optimum or premature convergence. Finally, non-convergence might appear when all fireflies share the same brightness.

A modified firefly optimization algorithm was developed to improve the search ability of the firefly optimization algorithm in this study. In the modified algorithm, the reverse learning strategy was introduced to initialize firefly individuals, the Rosenbrock search method was introduced to improve the search abilities of firefly individuals, and the Gaussian perturbation strategy, which is a kind of random disturbance with Gaussian distribution, was added in the process of particle swarm optimization to prevent the rapid convergence of the intelligent particle algorithm in mathematical operation, thus to prevent incorrect results and to enhance the group evolution ability. The modified search procedure could be described as follows:

Step 1: 

Initial population $x^{\left(1\right)}\in R^{n}$, which contains j decision individuals (number of firefly individuals); n refers to the dimension of the population variable. The unit orthogonal directions are $d^{\left(1\right)}$, $d^{\left(2\right)}$, ……, $d^{\left(n\right)}$; steps are $\delta_{1}^{\left(0\right)}$, $\delta_{2}^{\left(0\right)}$, ……, $\delta_{n}^{\left(0\right)}$; α refers to expansion factor where α>1, β refers to reduction factor where β∈(−1,0); ε refers to the allowable error where ε>0.

For i=1:n

$y^{\left(1\right)}=x^{\left(1\right)},\;k=1,\;j=1,\;\delta_{i}=\delta_{i}^{\left(0\right)}$

End for

Step 2:

If $f\left(y^{\left(j\right)}+\delta_{j}d^{\left(j\right)}\right)<f\left(y^{\left(j\right)}\right)$

$y^{\left(j+1\right)}=y^{\left(j\right)}$, $\delta_{j}=\alpha \cdot \delta_{j}$

Else

$y^{\left(j+1\right)}=y^{\left(j\right)}$, $\delta_{j}=\beta \cdot \delta_{j}$

End if

Step 3:

If j<n, then j=j+1, GO TO step 2; else GO TO step 4

Step 4:

If $f\left(y^{\left(n+1\right)}\right)<f\left(y^{\left(1\right)}\right)$, then $f\left(y^{\left(n+1\right)}\right)=f\left(y^{\left(1\right)}\right)$, GO TO step 2; If $f\left(y^{\left(n+1\right)}\right)=f\left(y^{\left(1\right)}\right)$, GO TO step 5.

Step 5:

If $f\left(y^{\left(n+1\right)}\right)<f\left(y^{\left(k\right)}\right)$, then GO TO step 6; else if $\left|\delta_{j}\right|\leq \varepsilon$ for all j, then stop searching, regard $x^{\left(k\right)}$ as the approximate optimal solution; otherwise $y^{\left(1\right)}=y^{\left(n+1\right)}$, j=1, GO TO step 2.

Step 6:

Make $x^{\left(k+1\right)}=y^{\left(n+1\right)}$, if $\mathrm{||}x^{\left(k+1\right)}-x^{\left(k\right)}\mathrm{||}<\varepsilon$, stop searching, regard $x^{\left(k+1\right)}$ as the approximate optimal solution; else GO TO step 7.

Step 7:

Make $x^{\left(k+1\right)}=x^{\left(k\right)}+\overset{n}{\underset{i=1}{\sum }}\lambda_{i}d^{\left(i\right)}$, where $\lambda_{i}=\overset{j}{\underset{1}{\sum }}d^{\left(i\right)}$. Define a set of direction vectors: $P^{\left(1\right)}$, $P^{\left(2\right)}$, ……, $P^{\left(n\right)}$:

$$P^{\left(j\right)}=\{\begin{array}{l} d^{\left(j\right)},\;\;\;\;\;\;\;\;\;\;\;\;\;\;\;\lambda_{j}=0\\ \overset{n}{\underset{i=j}{\sum }}\lambda_{i}d^{\left(i\right)}\;\;\;\;\;\lambda_{j}\neq 0 \end{array}$$

Normalize $\left\{P^{\left(j\right)}\right\}$ with Gram–Schmidt orthogonalization equation, make:

$$q^{\left(j\right)}=\{\begin{array}{l} P^{\left(j\right)},\;\;\;\;\;\;\;\;\;\;\;\;\;\;\;\;\;\;\;\;\;\;\;\;\;\;\;\;\;\;\;\;\;\;\;\;\;\;\;\;\;\;\;\;\;\;\;\;j=1\\ P^{\left(j\right)}-\overset{j-1}{\underset{i=1}{\sum }}\frac{q^{{\left(i\right)}^{T}}P^{\left(j\right)}}{q^{{\left(i\right)}^{T}}P^{\left(i\right)}}\cdot q^{\left(i\right)},\;\;\;\;\;\;\;\;\;\;\;\;\;\;j\geq 2 \end{array}$$

Conduct vector unitization and make $\bar{d^{\left(j\right)}}=\frac{q^{\left(j\right)}}{\mathrm{||}q^{\left(j\right)}\mathrm{||}}$, then *n* new orthogonal search directions will be determined.

Step 8: Make $d^{\left(j\right)}=\bar{d^{\left(j\right)}}$, $\delta_{j}=\delta_{j}^{\left(0\right)}$, j=1, 2, ……, n, $y^{\left(1\right)}=x^{\left(k+1\right)}$, k=k+1, j=1, GO TO step 2.

Based on the Rosenbrock search method, the Gaussian perturbation strategy was also introduced to reduce the premature phenomenon and keep the population diverse. The Gaussian perturbation strategy was only used on the optimal individual $x_{best}^{t}$ in the current population (the firefly population of the generation t) to increase the convergence rate of the modified firefly optimization algorithm in this study:

(7) $$G_{best}^{t}=x_{best}^{t}\cdot \left(1+Gaussian\left(\sigma \right)\right)$$

where: $G_{best}^{t}$ refers to the optimal individual in the firefly population of the generation t after the implementation of the Gaussian perturbation strategy, Gaussian(σ) is a random variable in Gaussian distribution.

The global optimal location is updated as:

(8) $$x_{best}^{t+1}=\{\begin{array}{c} G_{best}^{t}\;\;\;\;\;\;\;\;\;\;g\left(G_{best}^{t}\right)<g\left(x_{best}^{t}\right)\\ x_{best}^{t} \end{array}$$

where: g( ) refers to the fitness value of individuals.

It could be known from the Equation (7) that if the current optimal solution is the local optimal solution, the Gaussian perturbation used on the current optimal individual $x_{best}^{t}$ could effectively stop the algorithm from falling into the local optimum, improve the search efficiency of the Rosenbrock search method, and thus improve the traditional firefly optimization algorithm.

#### 2.2.2. The Firefly Asphalt Back Calculation Method (FABCM)

**Figure 2:**
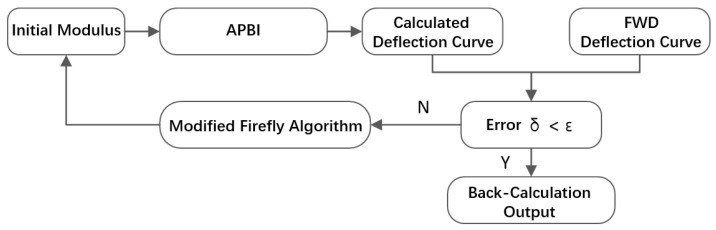
The Firefly Asphalt Back Calculation Method (FABCM) process.

Combined with the elastic layer system method (the program APBI) of road structure by FORTRAN, the modified firefly optimization algorithm introduced above is used to develop the asphalt pavement modulus back calculation program FABCM. The FABCM process is as shown in Figure 2:

#### 2.2.3. The Multi-Parameter FABCM

**Figure 3:**
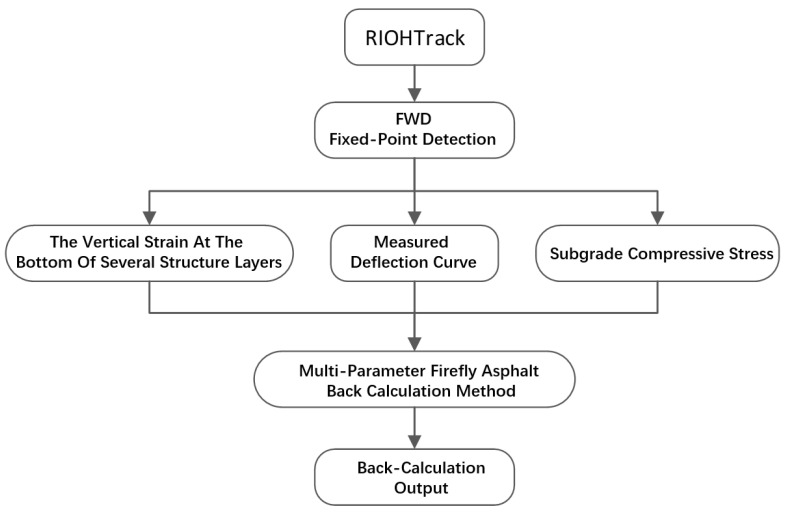
The multi-parameter FABCM process.

Popular asphalt pavement modulus back calculation methods used now are based only on the FWD deflection curve. Considering the unavoidable system errors in FWD deflection curve measurement, which could lead to inaccuracy, based on the FABCM introduced above, a multi-parameter firefly back calculation method with the vertical strain at the bottom of one or several of the structure layers, or with subgrade compressive stress as input parameters, was designed in this study. The process is as shown in Figure 3:

## 3. Results

### 3.1. The Verification of FABCM

**Figure 4:**
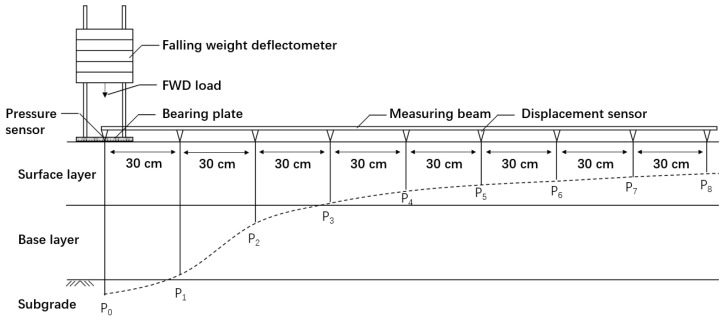
The distribution of deflection curve measuring points.

Asphalt pavement structure systems of three and four layers were selected to verify the stability and accuracy of FABCM, and the theoretical deflection curves of them were calculated with BISAR 3.0. The structure of the three layers consists of asphalt concrete surface, cement treated macadam base and soil subgrade, and the structure of the four layers shares the same materials, but with a cement treated soil subbase between the base and subgrade. The thicknesses and moduli of those structure layers are as shown in Table 3. After that, those calculated deflection curves were brought into FABCM as input parameters to conduct modulus back calculation. The applied load is a circular uniform load with a concentration of 0.707 MPa (the load concentration of standard axle load BZZ-100 in China) and a radius of 15 cm (usually used bearing plate size for FWD [42]). The distances between the deflection measuring points and load geometric center are: P_0_: 0 cm, P_1_: 30 cm, P_2_: 60 cm, P_3_: 90 cm, P_4_: 120 cm, P_5_: 150 cm, P_6_: 180 cm, P_7_: 210 cm, P_8_: 240 cm (as is shown in Figure 4). Poisson’s ratios are determined by 0.25 for surface material, 0.25 for base material, 0.35 for subbase material and 0.4 for subgrade material. The calculated theoretical deflection curves are as shown in Table 4.

The accuracy of back calculation is calculated by the Equations (9) and (10):

(9) $$\eta =\left|\frac{E-E_{0}}{E}\right|\times 100\%\;$$

(10) $$\delta =\left|\frac{W_{i}-D_{i}}{D_{i}}\right|\times 100\%$$

where $E_{0}$ refers to the theoretical modulus; E refers to the back calculated modulus; $D_{i}$ refers to the theoretical deflection curve; and $W_{i}$ refers to the back calculated reflection basin. The back calculation accuracy is as shown in Table 4:

The errors of back calculated modulus are all smaller than 1%, except for the subbase of structure 2, and that of the back calculated deflection curves, which are all smaller than 0.2%. Furthermore, FABCM will take only 0.5–1 s for each calculation. It could be proved that both the accuracy and calculation speed of FABCM are satisfactory.

### 3.2. Modulus Back Calculation Based on Theoretical Deflection Curves Calculated with BISAR3.0

To evaluate the influence of different axle loads and deflection measuring types on the modulus back calculation accuracy, four kinds of circular uniform loads and eight kinds of deflection types are used in the theoretical deflection curve calculation. Compared with the relatively fixed size of bearing plates in FWD tests (the circular plates with the radius of 15 cm are usually used [42]), the change of impact loads is more available, to give reasonable guidance for filed FWD tests, the change of load concentration rather than that of load size was used to simulate different axle loads. The four circular uniform loads are: ① Concentration of 0.707 MPa and radius of 15 cm (equivalent to the axle load of 5 t); ② Concentration of 0.990 MPa and radius of 15 cm (equivalent to the axle load of 7 t); ③ Concentration of 1.273 MPa and radius of 15 cm (equivalent to the axle load of 9 t); ④ Concentration of 1.556 MPa and radius of 15 cm (equivalent to the axle load of 11 t). The theoretical deflection curves of 6 asphalt pavement structures under four circular uniform loads were calculated with BISAR3.0. With eight different types of theoretical deflection curves (as shown in Figure 4 and Table 5), the modulus back calculation was conducted in FABCM.

#### 3.2.1. Back Calculation Errors of Surface Layer Modulus

**Figure 5:**
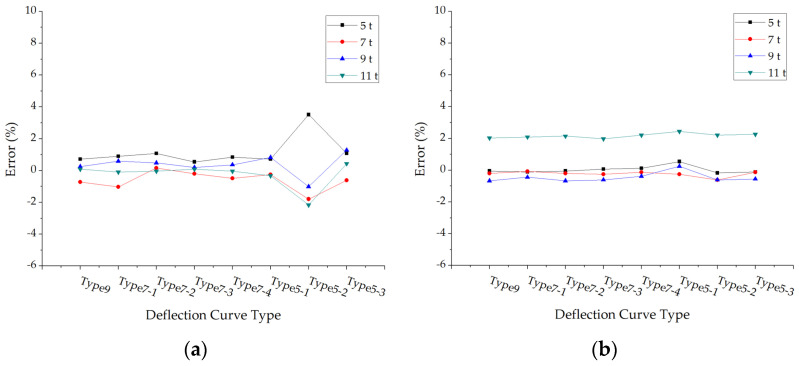

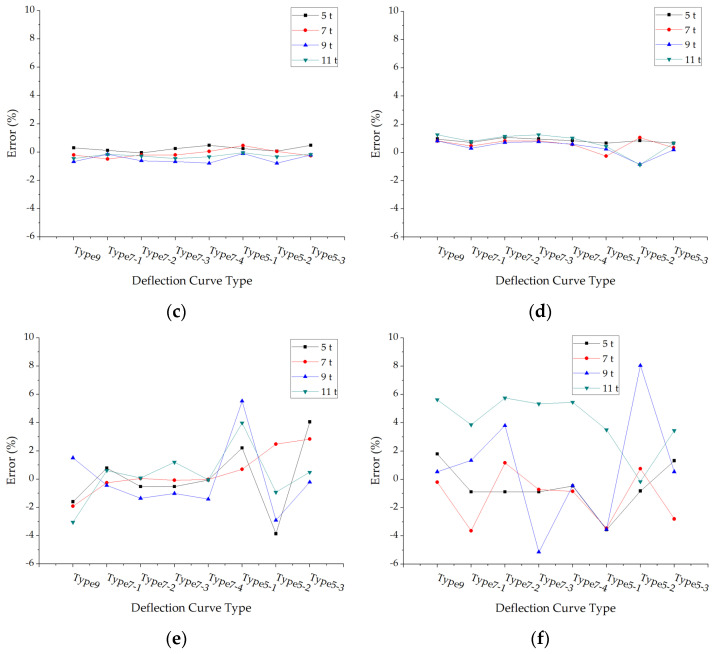
The errors of back calculated surface modulus under different deflection curve types: (**a**) Structure 1; (**b**) Structure 2; (**c**) Structure 3; (**d**) Structure 4; (**e**) Structure 5; (**f**) Structure 6.

The errors of back calculated surface modulus under different loads and pavement structures are shown in Figure 5.

The accuracies of this modulus back calculation method in each surface layer test are satisfying, and most of the errors are smaller than 2%. As for structure 5 and structure 6, the errors are relatively larger, but the maximum error is also smaller than 8%. Thus, the modulus back calculation results of asphalt pavement surface layers are reliable.

**Figure 6:**
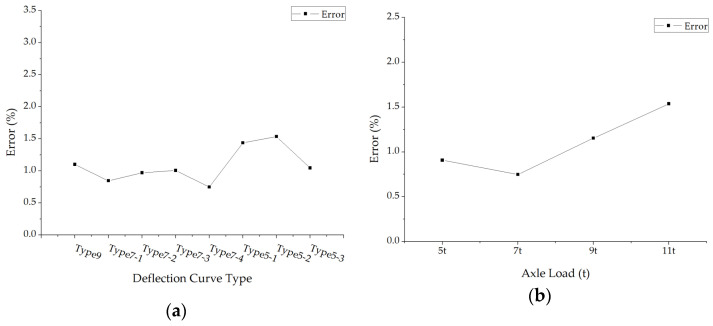
The average errors of back calculated surface modulus: (**a**) The average errors under different deflection curve types; (**b**) The average errors under different axle loads.

The average absolute values of surface modulus errors under different deflection curve types and axle loads were calculated and drawn in Figure 6. The average back calculation errors of the surface layer modulus fluctuate in a small amplitude under different forms of deflection curves, which means the accuracies of this new back calculation method are almost not affected by the forms of deflection curves. However, the errors decrease first and then rise with the increase in axle load.

#### 3.2.2. Back Calculation Errors of Base Modulus

**Figure 7:**
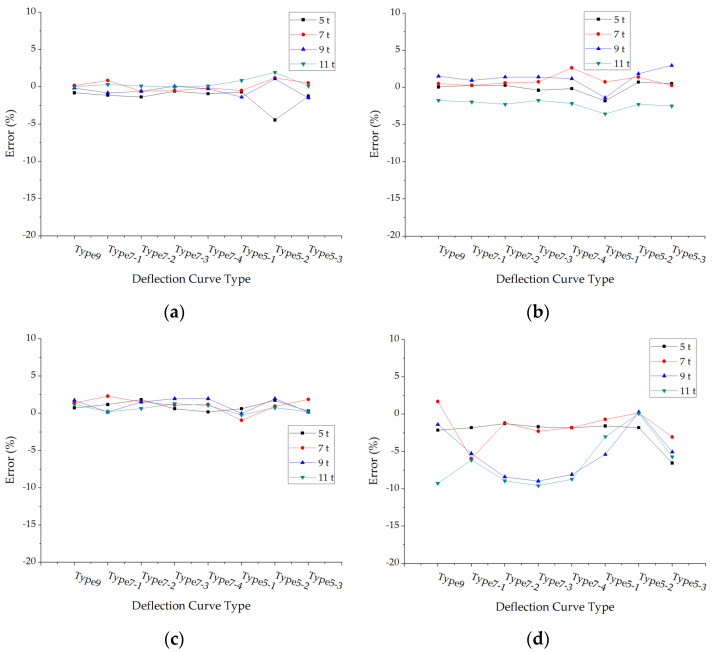

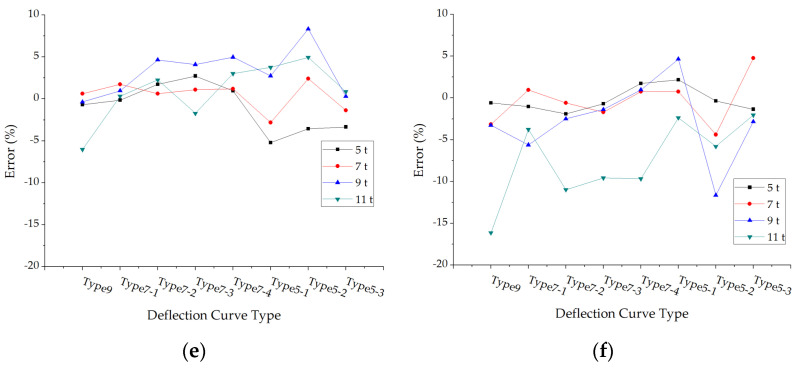
The errors of back calculated base modulus under different deflection curve types: (**a**) Structure 1; (**b**) Structure 2; (**c**) Structure 3; (**d**) Structure 4; (**e**) Structure 5; (**f**) Structure 6.

**Figure 8:**
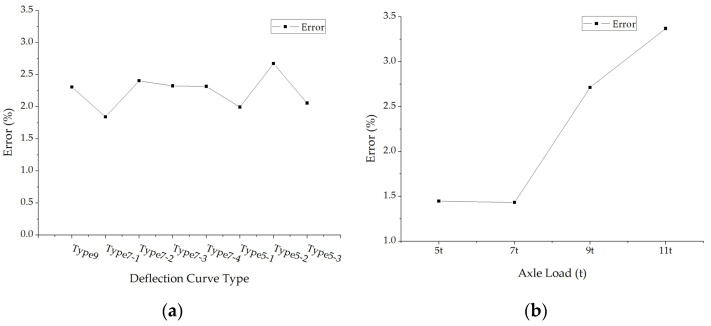
The average errors of back calculated base modulus: (**a**) The average errors under different deflection curve types; (**b**) The average errors under different axle loads.

The errors of back calculated base modulus under different loads and pavement structures are shown in Figure 7. The average absolute values of base modulus errors of each structure under different deflection curve types and axle loads were calculated and drawn in Figure 8.

It could be found from Figure 7 that the back calculation errors under each structure are basically within 2%, but the errors of structure 5 and structure 6 are larger than others. The error of structure 6 under the deflection curve type 9 and the axle load of 11 t even exceeds 15%. Compared with the surface layer, the modulus back calculation errors of the base layer are larger in general.

Figure 8a is the relation between the errors of back calculated base modulus and deflection curve types and this is still unclear. Whereas, it is clear that the errors are influenced by axle loads, as when the axle load increased into 11 t the error is as double what it would be in 5 t or 7 t (shown as Figure 8b) and this trend is similar with the one in Figure 6.

#### 3.2.3. Back Calculation Errors of Subbase Modulus

**Figure 9:**
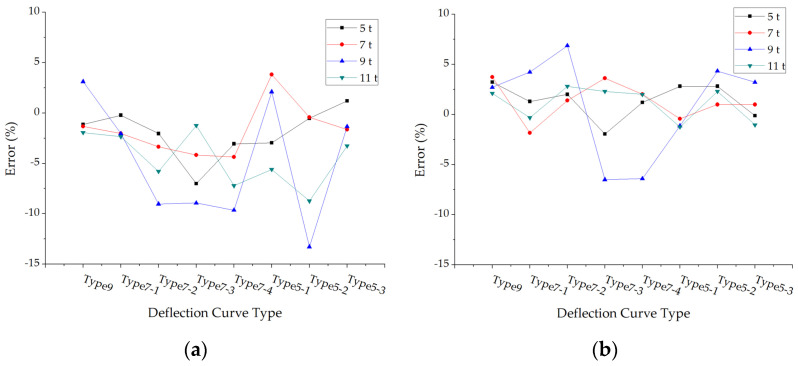
The errors of back calculated subbase modulus under different deflection curve types: (**a**) Structure 5; (**b**) Structure 6.

**Figure 10:**
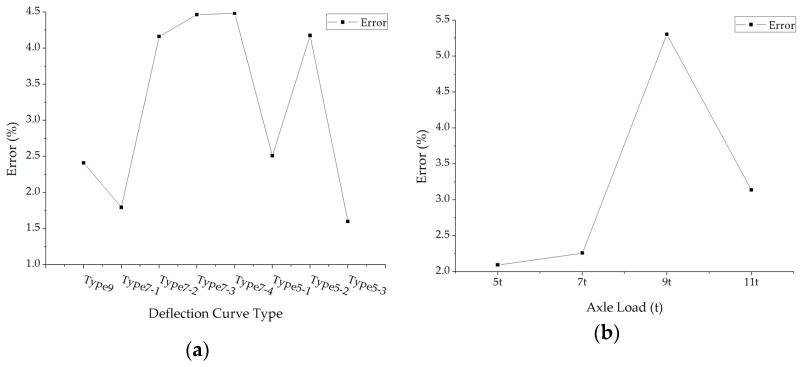
The average errors of back calculated subbase modulus: (**a**) The average errors under different deflection curve types; (**b**) The average errors under different axle loads.

Only structure 5 and structure 6 possess subbase layers, the errors of back calculated subbase modulus under different loads and two pavement structures are shown in Figure 9. The average absolute values of the subbase modulus errors of each structure under different deflection curve types and axle loads were calculated and shown in Figure 10.

The most errors in Figure 9 are within 10%, while the errors fluctuate with a big amplitude for both deflection curve types and axle loads, thus, Figure 10 shows no evident rules. A similar trend is also found in the subbase layer, which is where modulus errors increase sharply after a 7 t axle load. 

#### 3.2.4. Back Calculation Errors of Subgrade Modulus

**Figure 11:**
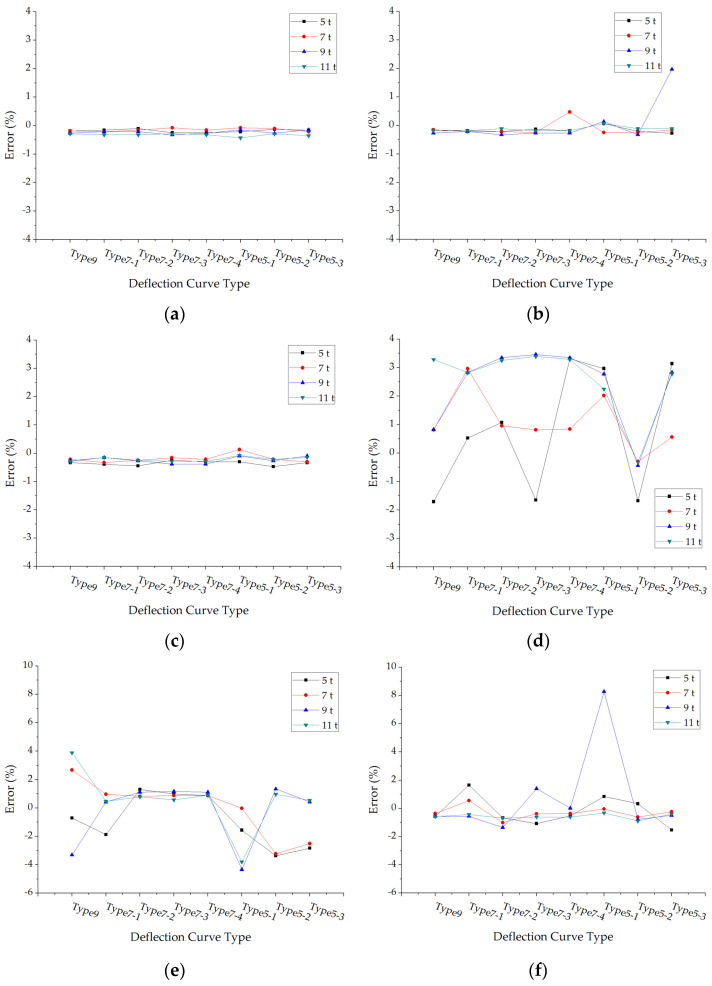
The errors of back calculated subgrade modulus under different deflection curve types: (**a**) Structure 1; (**b**) Structure 2; (**c**) Structure 3; (**d**) Structure 4; (**e**) Structure 5; (**f**) Structure 6.

**Figure 12:**
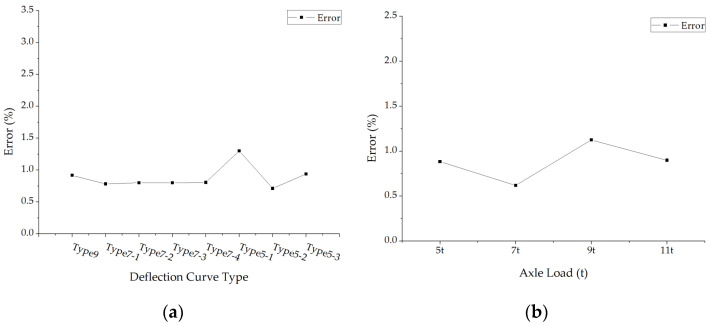
The average errors of back calculated subgrade modulus: (**a**) The average errors under different deflection curve types; (**b**) The average errors under different axle loads.

The errors of back calculated subgrade modulus under different loads and pavement structures are shown in Figure 11. The average absolute values of subgrade modulus errors with each structure case under different deflection curve types and axle loads were calculated and shown in Figure 12.

The back calculation errors of subgrade modulus are generally smaller, which means the accuracy of back calculated subgrade modulus is higher. It could be seen from Figure 12 that the errors of back calculated subgrade modulus have few effects by the deflection curve types and axle load levels.

### 3.3. Modulus Back Calculation Based on Actual Deflection Curves Measured on RIOHTrack

**Figure 13:**
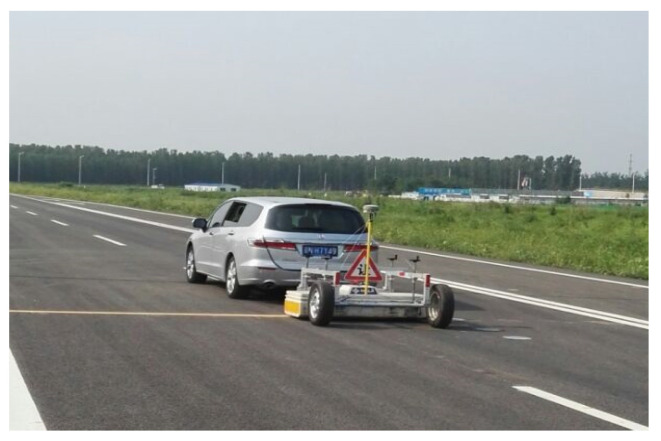
The MIRA.

Considering the structure layer thickness as one of the most important parameters, which influences the modulus back calculation results greatly, it is important to measure the thickness of each layer in the four selected structures. The Mala Imaging Radar Array (MIRA) manufactured by Swedish MALA company was used to measure the thicknesses precisely, as is shown in Figure 13.

**Figure 14:**
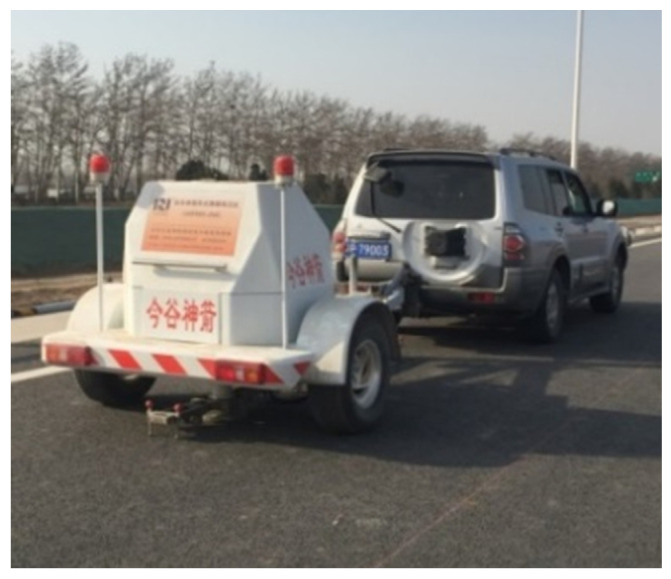
The FWD equipment.

According to the error analysis in Section 3.2, it could be known that there is no clear connection between modulus back calculation errors and deflection curve types, which indicates that the modulus back calculation accuracy is hardly influenced by the type of deflection curve. Based on this conclusion, considering that the deflection curve shape cannot be accurately described when the number of measuring points is too small, which could lead to the inaccuracy of modulus back calculation, the deflection curve type 9 was used in FWD tests due to its abundant measuring points. The FWD equipment used in this study is shown in Figure 14. The concentrated loads in FWD tests are 5 t, 7 t, 9 t and 11 t.

The modulus back calculation results are shown in Table 6, Table 7 and Table 8.

The average back calculated moduli of subgrades are all around 300 MPa, the average standard deviations are around 100, and the average coefficients of variation are generally smaller than that of asphalt surface layers and middle layers. Both the average standard deviations and the average coefficients of variation in simplified middle layers are big, where the average standard deviations are all bigger than 3500, and the coefficients of variation are between 52% and 106%. This could be easily explained by the material diversity of middle layers. The back calculated moduli of asphalt concrete layers possess the biggest average standard deviation, but the average coefficients of variation are still between 60% and 83%, which means the dispersion of modulus back calculation of asphalt concrete surface is more stable than that of middle layers.

### 3.4. Modulus Back Calculation with Multi-Parameter FABCM

To test the reliability and accuracy of multi-parameter FABCM, and compare it with single-parameter FABCM, the vertical pressure on the top of the subgrade was used with a measured deflection curve together to conduct modulus back calculation.

When the RIOHTrack was constructed, different sensors including strain sensors, stress sensors, temperature monitors, hygrometers, etc., were buried in the structures. Hence, when different loads are applied on the road surface, the strain and stress states inside the pavement structures could be determined with data from those sensors. There are vertical stress sensors installed in the top-surface of the subgrade of each pavement structure, therefore, it is possible to know the vertical pressure on the top surface of the subgrade when loads are applied on the road, and the modulus back calculation based on the multi-parameter method is capable with those stress values.

The multi-parameter modulus back calculation was only performed on STR1 and STR19 for their relatively simple structures. The simplified structures are shown in Table 2, and the measured vertical pressure on the top surfaces of subgrades under different loads are shown in Table 9.

**Figure 15:**
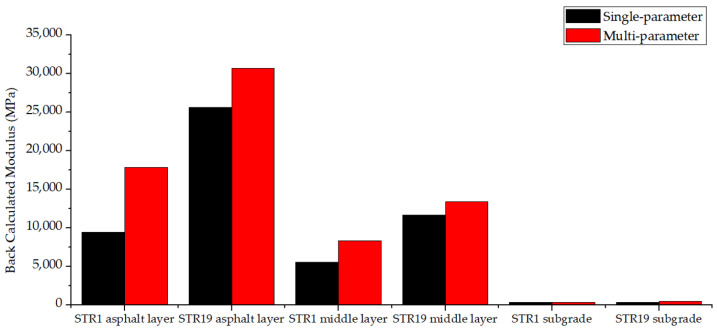
The back calculated modulus.

**Figure 16:**
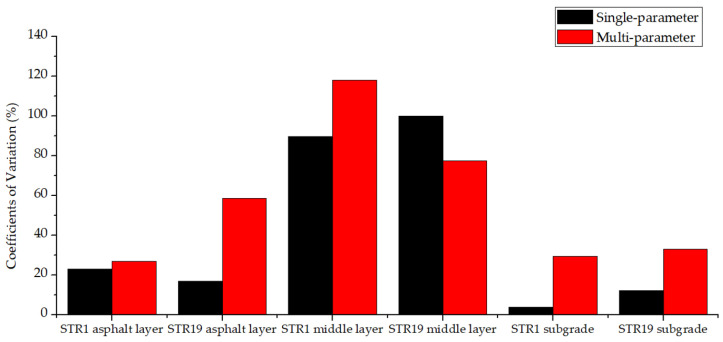
The coefficients of variation.

To compare the single-parameter FABCM and the multi-parameter FABCM, the back calculation results and the coefficients of variation in two methods are shown in Figure 15 and Figure 16.

For the same structure layer, the back calculated modulus of multi-parameter FABCM differs greatly from that of single-parameter FABCM. The differences vary with different structure layers, and few relations could be found. When it comes to the coefficients of variation, it could be seen from Figure 16 that the variation coefficient of multi-parameter FABCM results is usually larger than that of single-parameter FABCM results for the same structure layer. Both of the two methods provide relatively stable modulus back calculation results for asphalt layers and the subgrade, while the results for the middle layers possess larger variation coefficients.

## 4. Discussion

The accuracy of this novel modulus back calculation is influenced greatly with pavement structures. When the pavement structure consists of three layers and the asphalt concrete surface layer is thin, like the structure 1, structure 2 and structure 3 in Section 3.2, the accuracy will be the highest. The iteration termination condition of back calculation is that $\left|\delta_{j}\right|\leq \varepsilon$, therefore, the final output modulus combination of the back calculation program is the optimal value of the mathematical optimization algorithm, rather than the modulus combination with the smallest error. When the number of pavement structure layers increase, more modulus and thickness parameters will be needed to be matched. The increase in the number of matching parameters increases the probability of the phenomenon mentioned above, which could lead to the inaccuracy of modulus back calculation. Thus, the smaller the number of structure layers, the higher the modulus back calculation accuracy.

For the subgrade material, the differences between the back calculated moduli of STR1, STR18 and STR19 gradually increase with the increase in the axle load. Under the same axle load, the back calculated modulus of STR10 is the largest. It shows that the subgrade material has typical nonlinear mechanical characteristics, and its back calculated modulus depends not only on the pavement structure, but also on the axle load applied on the road surface.

As for the middle layers, it could be seen that whatever the base type is, the back calculated modulus would be influenced by the level of axle load. Compared with that of flexible base and composite base, this influence on the modulus of semi-rigid base is not obvious.

When it comes to the asphalt layers, the back calculated modulus increases first and then decreases with the increase in axle load and finally tends to be stable, which is affected by the structure form. The thicker the asphalt layers are, the greater the load that the middle layer can bear, and the bigger the back calculated modulus of the asphalt layer would be.

The essence of the modulus back calculation is the match between the measured deflection curve and the theoretical deflection curve under the elastic layer system theory. Since FWD can be regarded as an instantaneous impact load (generally about 0.02 s), the response state of the pavement structure will also have nonlinear characteristics under the impact load of FWD due to the typical nonlinear characteristics of pavement materials. Therefore, no matter what kind of optimization algorithm is used in the modulus back calculation, the actual measured deflection curve is quite difficult to be consistent with the theoretical deflection curve under the current elastic mechanical model, which is also the reason that the back calculated modulus is not consistent with the design modulus of the pavement structure layer.

As is verified in Section 3.1, when the back calculation was conducted based on the theoretical deflection curves, which means there were no error caused by the differences between elastic mechanical model and non-linear characteristics of asphalt pavement materials, both the accuracy and calculation speed of FABCM are satisfactory. It could be told that the inherent defects of the firefly optimization algorithm could be avoided effectively with the modifying methods in this paper, while the calculation speed is not obviously affected.

The variation coefficient of multi-parameter FABCM results is larger than that of the single-parameter FABCM, which means in actual practice, the result of single-parameter FABCM is more reliable than that of multi-parameter FABCM due to the acquisition and accumulation errors of the input parameters in the multi-parameter FABCM program. Besides, compared with the deflection curve, the stress and strain inside the pavement structures are usually harder to be precisely measured, thus the single-parameter back calculation method is more recommended.

## 5. Conclusions

In this study, an asphalt pavement modulus back calculation method based on the firefly optimization algorithm was proposed to investigate the factors influencing back calculation accuracy and the way to select proper input parameters for modulus back calculation. The main findings are summarized as follows:

1. The firefly algorithm, a meta-heuristic intelligent optimization algorithm, is selected as the mathematical search method of the modulus back calculation algorithm, and the Rosenbrock search method and Gaussian perturbation strategy are used to improve the firefly algorithm in terms of search rules and intermediate optimal solution disturbance. Through a lot of calculation and comparative analysis, it is verified that FABCM has a satisfactory back calculation speed and accuracy;
2. There is no clear connection between the modulus back calculation errors and deflection curve types, which indicates that the modulus back calculation accuracy is not influenced by the type of deflection curve. Thus, considering that the deflection curve shape cannot be accurately described when the number of measuring points is too small, which could lead to the inaccuracy of the modulus back calculation, the deflection curve types of more measuring points are recommended in modulus back calculation;
3. It is found that when the axle load reaches 9 t or 11 t, the errors of back calculated modulus would increase a lot, and the back calculation on the 3-layer structure is more accurate than that on the 4-layer structure;
4. Due to the significant non-linear characteristics of pavement materials, no matter what kind of optimization algorithms are used, the back calculated modulus is hard to be consistent with the design modulus of the pavement structure layer under the current elastic mechanical model;
5. The variation coefficients of multi-parameter FABCM are generally larger than that of single-parameter FABCM in practice, which means the results of single-parameter FABCM are more stable. Considering the reasons discussed in Section 4, the single-parameter modulus back calculation method has its advantages in measurement of stress and strain state inside a pavement structure and shows potential application prospects.

The asphalt pavement mechanical model in FABCM is still an elastic model, which could not describe the non-linear characteristics of realistic asphalt pavement materials. Considering that the inherent defects, such as premature convergence and the local optimum of mathematical optimization algorithm, were effectively avoided with a Rosenbrock search method and Gaussian perturbation strategy in this study, the main reason why great errors occur when conducting modulus back calculation based on the measured deflection curves on RIOHTrack should be the difference between an elastic mechanical model and the non-linear asphalt pavement characteristics. To achieve the best possible match between the design modulus and the back calculated modulus in the future, we recommend the study and use of non-linear mechanical models, which could describe the asphalt pavement characteristics better in the back calculation methods.

## Figures and Tables

**Table 1:** Material and structure parameters.

Structure Number	Material	Thickness (cm)	Modulus (MPa)	Poisson’s Ratio
1	Asphalt concrete	18	2000	0.25
	Cement treated macadam	40	1700	0.25
	Soil base	/	40	0.4
2	Asphalt concrete	36	2000	0.25
	Graded macadam	40	300	0.35
	Soil base	/	40	0.4
3	Asphalt concrete	36	2000	0.25
	Graded macadam	40	300	0.35
	Soil base	/	60	0.4
4	Asphalt concrete	48	2000	0.25
	Cement treated macadam	40	1700	0.25
	Soil base	/	40	0.4
5	Asphalt concrete	24	2000	0.25
	Graded macadam	30	300	0.35
	Cement treated soil	30	600	0.25
	Soil base	/	40	0.4
6	Asphalt concrete	18	2000	0.25
	Cement treated macadam	20	1700	0.25
	Cement treated soil	20	600	0.25
	Soil base	/	40	0.4

The parameters were obtained from project design documentations.

**Table 2:** The simplified 4 structures on RIOHTrack.

**Layer**	**STR1**			**STR10**		
	**Modulus** **(MPa)**	**Poisson’s** **Ratio**	**Thickness** **(m)**	**Modulus** **(MPa)**	**Poisson’s** **Ratio**	**Thickness** **(m)**
1	9620	0.25	0.52	6300	0.25	0.28
2	2000	0.25	0.4	900	0.3	0.2
3	120	0.35		3250	0.25	0.4
4				120	0.35	
**Layer**	**STR18**			**STR19**		
	**Modulus** **(MPa)**	**Poisson’s** **Ratio**	**Thickness** **(m)**	**Modulus** **(MPa)**	**Poisson’s** **Ratio**	**Thickness** **(m)**
1	8830	0.25	0.48	13,200	0.25	0.48
2	900	0.3	0.48	6000	0.25	0.2
3	120	0.35		120	0.35	

The parameters were obtained from the documentations of RIOHTrack, and the elastic moduli of asphalt layers are under 20 °C, as is specified in Specifications for Design of Highway Asphalt Pavement [36].

**Table 3:** The theoretical deflection curves calculated in BISAR3.0.

	Thickness (cm)	Modulus (MPa)	Theoretical Deflection Value (0.01mm)								
			0	30	60	90	120	150	180	210	240
			P_0_	P_1_	P_2_	P_3_	P_4_	P_5_	P_6_	P_7_	P_8_
1	18/40	1800/1500/40	46.3	36.8	32.6	29.1	25.8	22.8	20.2	17.9	15.9
2	18/40/20	1800/1500/600/40	40.7	31.5	28.2	25.6	23.2	20.9	18.9	17.0	15.4

**Table 4:** The modulus back calculation accuracy.

	Back Calculated Modulus (MPa)	*η* (%)	*δ* (%)								
			*δ*_0_	*δ*_1_	*δ*_2_	*δ*_3_	*δ*_4_	*δ*_5_	*δ*_6_	*δ*_7_	*δ*_8_
1	1802.0/1485.4/40.0	0.11/0.97/0.14	0.06	0.08	0.04	0.02	0.05	0.07	0.08	0.06	0.04
2	1809.7/1488.5/608.8/39.6	0.54/0.77/1.44/0.98	0.07	0.12	0.01	0.07	0.09	0.13	0.14	0.09	0.07

**Table 5:** The measuring points configurations of eight types of deflection curves.

Deflection Curve Type	Deflection Measuring Points
9	P_0_, P_1_, P_2_, P_3_, P_4_, P_5_, P_6_, P_7_, P_8_
7−1	P_0_, P_1_, P_2_, P_3_, P_4_, P_5_, P_6_
7−2	P_0_, P_1_, P_2_, P_3_, P_4_, P_6_, P_8_
7−3	P_0_, P_1_, P_2_, P_3_, P_4_, P_7_, P_8_
7−4	P_0_, P_1_, P_2_, P_4_, P_6_, P_7_, P_8_
5−1	P_0_, P_1_, P_2_, P_3_, P_4_
5−2	P_0_, P_2_, P_4_, P_6_, P_8_
5−3	P_0_, P_1_, P_2_, P_4_, P_6_

**Table 6:** The modulus back calculation results of subgrade.

	**5 t**			**7 t**		
	**Average** **Modulus (MPa)**	**Standard** **Deviation**	**Coefficient of** **Variation (%)**	**Average** **Modulus (MPa)**	**Standard** **Deviation**	**Coefficient of** **Variation (%)**
STR1	275.03	29.3	11	294.63	37.3	13
STR10	359.78	137.85	38	405.67	193.46	48
STR18	265.26	191.81	72	281.56	77.77	28
STR19	257.51	29.39	11	320.84	177.39	55
Average	289	97	33	325	121	36
	**9 t**			**11 t**		
	**Average** **Modulus (MPa)**	**Standard** **Deviation**	**Coefficient of** **Variation (%)**	**Average** **Modulus (MPa)**	**Standard** **Deviation**	**Coefficient of** **Variation (%)**
STR1	273.1	20.47	7	276.35	24.2	9
STR10	320	60.81	19	375.86	90.38	24
STR18	308.65	88.32	29	356.73	204.07	57
STR19	243.3	9.44	4	280.17	116.8	42
Average	286.26	45	15	322	108	33

**Table 7:** The modulus back calculation results of middle layer.

	**5 t**			**7 t**		
	**Average** **Modulus (MPa)**	**Standard** **Deviation**	**Coefficient of** **Variation (%)**	**Average** **Modulus (MPa)**	**Standard** **Deviation**	**Coefficient of** **Variation (%)**
STR1	1702.55	1184.1	70	12,781	15,404.8	121
STR10 (1)	7455.04	3940.32	53	11,094	8477.23	76
STR10 (2)	13,726.8	14,815.3	108	4123.7	7077.58	172
STR18	1013.07	245.89	24	1020.9	347.57	34
STR19	4326.61	943.56	22	28,499	36,864.8	129
Average	5645	4225	55	11,504	13634	106
	**9 t**			**11 t**		
	**Average** **Modulus (MPa)**	**Standard** **Deviation**	**Coefficient of** **Variation (%)**	**Average** **Modulus (MPa)**	**Standard** **Deviation**	**Coefficient of** **Variation (%)**
STR1	3713.2	3047.01	82	3926.1	3262.34	83
STR10 (1)	8514.3	4090.67	48	9902.3	3996.46	40
STR10 (2)	10,365	5510.28	53	2732	1248.84	46
STR18	820.28	268.94	33	718.84	353.56	49
STR19	10,456	5109.43	49	3445.7	1464.08	42
Average	6774	3605	53	4145	2065	52

**Table 8:** The modulus back calculation results of asphalt concrete surface layer.

	**5 t**			**7 t**		
	**Average** **Modulus (MPa)**	**Standard** **Deviation**	**Coefficient of** **Variation (%)**	**Average** **Modulus (MPa)**	**Standard** **Deviation**	**Coefficient of** **Variation (%)**
STR1	6259	2748.5	44	10,625	12,632	119
STR10	19,564	14,164	72	20,200	14,622	72
STR18	15,688	10,266	65	17,937	12,517	70
STR19	19,978	15,288	77	30,391	21,156	70
Average	15,372	10,616	65	19,788	15,231	83
	**9 t**			**11 t**		
	**Average** **Modulus (MPa)**	**Standard** **Deviation**	**Coefficient of** **Variation (%)**	**Average** **Modulus (MPa)**	**Standard** **Deviation**	**Coefficient of** **Variation (%)**
STR1	10,959	6709	61	9697	6828	70
STR10	24,145	12,039	50	15,382	12,352	80
STR18	20,513	11,914	58	23,305	11,846	51
STR19	26,761	19,608	73	25,335	16,451	65
Average	20,594	12,567	60	18,430	11,869	66

**Table 9:** The measured vertical pressures.

Structure	Vertical Pressure on the Top of Subgrade (kPa)			
	5 t	7 t	9 t	11 t
STR1	0.198	0.288	0.336	0.54
STR19	0.25	0.344	0.438	0.625

## Data Availability

Not applicable.
